# Global access of rifabutin for the treatment of tuberculosis – why should we prioritize this?

**DOI:** 10.1002/jia2.25333

**Published:** 2019-07-18

**Authors:** Neesha Rockwood, Maddalena Cerrone, Melissa Barber, Andrew M Hill, Anton L Pozniak

**Affiliations:** ^1^ Department of Medicine Imperial College London London UK; ^2^ Department of HIV Medicine Chelsea and Westminster Hospital London UK; ^3^ Department of Global Health and Population Harvard TH Chan School of Public Health Boston MA USA; ^4^ Department of Pharmacology and Therapeutics Liverpool University Liverpool UK; ^5^ Department of Clinical Research London School of Tropical Medicine and Hygiene London UK

**Keywords:** tuberculosis, HIV, Rifabutin, antiretroviral therapy, treatment outcomes, pharmacokinetic interactions, toxicity, switch, drug‐resistant‐TB, cost effectiveness

## Abstract

**Introduction:**

Rifabutin, a rifamycin of equivalent potency to rifampicin, has several advantages in its pharmacokinetic and toxicity profile, particularly in HIV co‐infected patients on combined antiretroviral therapy (cART). In this commentary, we evaluate evidence supporting increased global use of rifabutin and highlight key recommendations for action.

**Discussion:**

Although extrapolation of data from HIV uninfected patients would suggest non‐inferiority, there has been no randomized controlled study comparing rifabutin versus rifampicin in the outcomes of relapse‐free cure, in drug susceptible tuberculosis (TB), in HIV co‐infected patients on currently utilized cART regimens or in paediatric populations. An important advantage of rifabutin is that compared to the dose adjustments required with rifampicin, it can be co‐administered with the integrase strand transfer inhibitors raltegravir or dolutegravir without the need for dose adjustments. This strategy would be easier to implement in a programmatic setting and would save costs. We have assessed cost incentives to utilize rifabutin and have estimated generic costs for a range of rifabutin dosage scenarios. Where facilities are present for drug re‐challenge and monitoring for drug toxicity and cross‐reactivity, rifabutin offers a switch alternative for adverse drug reactions (ADR)s attributed to rifampicin. This would negate the need to prolong treatment in the absence of a rifamycin as part of short‐course multidrug therapy. There is evidence of incomplete cross‐resistance to rifampicin and rifabutin. Rifabutin may be useful in rifampicin‐resistant TB, in an estimated 20% of cases, based on phenotypic or genotypic rifabutin susceptibility testing.

**Conclusions:**

Rifabutin should be available globally as a first‐line rifamycin in HIV co‐infected individuals and as a switch option in cases of rifampicin associated ADRs. Further studies are needed to ascertain the utility of rifabutin in rifampicin‐resistant rifabutin‐susceptible TB.

## 1 Introduction

In programmatic settings where those managing medications may not be specialists, it is of vital importance to minimize complexity of antiretroviral drug dose adjustments in regimens for HIV‐tuberculosis (HIV‐TB) co‐infection. The purpose of this commentary is to advocate for widespread access to the option of rifabutin, as a first‐line rifamycin in the treatment of TB, especially in HIV co‐infected individuals.

Treatment outcomes, pharmacokinetic data, dosing recommendations, toxicity and switch data will be summarized and discussed. Utility in rifampicin‐resistant rifabutin‐susceptible TB and cost considerations will be reviewed. We will highlight key knowledge gaps and recommendations for action.

Rifampicin is important in the Short‐Course Chemotherapy regimen for drug‐susceptible TB, because of its potent bactericidal and sterilizing activity, to ensure relapse‐free cure 1. Rifampicin is a potent inducer of the nuclear pregnane X receptor and constitutive androstane receptor which leads to activation of target genes including enzymes such as hepatic cytochrome P450 system, p‐glycoprotein and glucuronosyltransferases 2. The consequence of this induction is a significant drug‐drug interaction potential and, for example, the need to dose adjust or avoid several antiretroviral agents.

Rifabutin is a rifamycin, which like rifampicin, works via inhibition of DNA‐dependent RNA synthesis in prokaryotes. Rifabutin has a high volume of distribution, concentrates well in the lung and has high intracellular penetration 3. It has an active metabolite, 25‐*O*‐desacetyl rifabutin (des‐rifabutin). Importantly, des‐rifabutin induces CYP3A and glucuronosyltransferases significantly less than rifampicin 4, 5 and so have fewer significant drug‐drug interactions. As a result of this property, rifabutin was included by the WHO in 2009 in the List of Essential Medicines for treatment of TB in patients with HIV receiving boosted protease inhibitors (bPIs). Until 2014, however, when rifabutin came off patent, widespread access was prohibited by cost.

## 2 Treatment outcomes

Two randomized controlled studies (RCTs) in HIV uninfected patients with pulmonary TB showed no significant differences in cure (RR 1.00, 95% CI 0.96 to 1.04; 553 participants) and relapse (RR 1.23, 95% CI 0.45 to 3.35; 448 participants) rates comparing rifabutin‐ and rifampicin‐containing treatment 6. Treatment outcomes for rifampicin versus rifabutin have been reported from two United Kingdom‐based retrospective observational studies of HIV co‐infected individuals. Singh *et al*. (n = 141, 75% on combined antiretroviral therapy (cART) during treatment) showed no significant differences in completion and relapse rates. Rawson *et al*. (n = 171) showed higher rates of completion with rifampicin than with rifabutin, with no difference in mortality or relapse rates. Schwander *et al*. (n = 50, 0% on cART) reported a faster rate of sputum smear conversion with rifabutin versus rifampicin (*p* < 0.05, log rank test) 7. Multiple studies have shown increased rates of acquired drug resistance with intermittent dosing both for rifampicin and rifabutin 8, 9, 10, 11, 12, particularly in those with advanced immunosuppression and disseminated bacillary burden 9, 10, 11, 13. There has been no evidence of increased risk of acquired rifabutin monoresistance in HIV co‐infected individuals when rifabutin is dosed daily and hence, this is recommended, subject to pharmacokinetic considerations.

Although extrapolation of data from HIV uninfected patients would suggest non‐inferiority of rifabutin versus rifampicin in relapse‐free cure in drug‐susceptible TB, there are no RCTs comparing treatment outcomes in HIV co‐infected patients. A current research gap is lack of a non‐inferiority RCT comparing daily rifampicin versus rifabutin in HIV co‐infected patients taking current standard of care cART regimens. There are also limited efficacy, pharmacokinetic and safety data from rifabutin studies in paediatric populations.

## 3 Pharmacokinetic data and dosing considerations with antiretroviral drugs

The recommended dose of rifabutin in drug susceptible TB is 5 mg/kg. Weiner *et al*. have suggested a threshold of area under the concentration time curve (AUC_0‐24 hr_) and peak concentration (C_max_) for rifabutin of 4.5 mg hr/L and 0.45 mg/L, respectively, for prevention of failure/relapse with acquired rifamycin resistance 14. Concurrent administration with food leads to delayed absorption and a mild (<20%) decrease in C_max_ of rifabutin. There is no significant effect of food on AUC_0‐24 hr_ for rifabutin and hence, it can be taken with or without food 15. An important barrier to the use of rifabutin is the lack of a fixed dose combination with other first‐line anti‐TB medications to optimize programmatic care.

Choice of first‐line rifamycin in HIV co‐infected individuals should ensure minimal toxicity and drug‐drug interactions in patients commencing and being maintained on cART. Table 1 summarizes interactions and dosing considerations with rifabutin and co‐formulated cART regimens.

**Table 1:** Interactions and dosing considerations with rifabutin and co‐formulated cART regimens

cART regimen	Drug‐drug interaction with rifabutin 300 mg od	Recommendation for co‐administration with rifabutin
Integrase strand transfer inhibitor		
Triumeq^®^ (dolutegravir/abacavir/Lamivudine)	Nil significant	No dose adjustment
Biktarvy^®^ (bictegravir/emtricitabine/tenofovir alafenamide)	Bictegravir C_max_, AUC and C_min_ are decreased by 20%, 38% and 56%* 50	Co‐administration not recommended due to potential suboptimal bictegravir levels
Stribild^®^ (elvitegravir/emcitrabine/tenofovir disoproxil fumarate) Genvoya^®^ (elvitegravir/emcitrabine/tenofovir alafenamide)	Elvitegravir AUC and C_min_ are decreased by 21% and 67%. des‐rifabutin exposures are increased by 4.8 to 6.3 fold* 51	Co‐administration not recommended due to potential suboptimal elvitegravir levels and rifabutin‐associated toxicity
Raltegravir + 2NRTI	Nil significant	No dose adjustment
Boosted protease inhibitor (bPI)		
Atazanavir/ritonavir + 2NRTI Darunavir/ritonavir + 2NRTI Lopinavir/ritonavir + 2NRTI	When co‐administered with bPI, rifabutin and des‐rifabutin exposures are significantly increased des‐rifabutin AUC increased over 10 fold) 52 Darunavir AUC is increased 50% to 60% 52	To minimize chances of acquired drug resistance, the US guidelines recommend once daily dosing of rifabutin 150 mg, along with enhanced monitoring for rifabutin‐related toxicity. No dosage change in bPI is recommended
Evotaz^®^ (atazanavir/cobicistat) + 2NRTI Rezolsta^®^ (daruvavir/cobicistat) + 2NRTI	Co‐administration of cobicistat with atazanavir or darunavir has not been studied but cobicistat levels may be reduced, thereby reducing atazanavir and darunavir levels. Des‐rifabutin levels are also likely to be significantly increased	European guidelines recommend if combination is needed, administer cobicistat bPI ×3/week with additional monitoring for des‐rifabutin associated toxicity
Non‐nucleotide reverse transcriptase inhibitor		
Atripla^®^ (efavirenz/emcitrabine/tenofovir disoproxil fumarate)	Rifabutin AUC, C_max_ and C_min_ decrease by 38%, 32% and 45% 21	Increase rifabutin to 450 mg od
Nevirapine + 2NRTI	Nil significant	Nil dose adjustment
Eviplera^®^ (rilpivirine/emcitrabine/tenofovir disoproxil fumarate) Odefsey^®^ (rilpivirine/emcitrabine/tenofovir alafenamide)	Rilpivirine C_max_, AUC and C_min_ reduced by 31%, 42% and 48% When compared to rilpivirine 25 mg od, coadminstration of rifabutin 300 mg and rilpivirine 50 mg increased AUC and C_max_ by 16% and 43% respectively* 52	Co‐administration not recommended according to US guidelines due to suboptimal rilpivirine levels. European guidelines recommend an additional 25 mg dose of rilpivirine once daily
Etravarine + 2NRTI	Nil significant 53	No dose adjustment if not co‐administered with bPI Concomitant bPI with etravirine and rifabutin is not recommended due to expected additional decrease in etravirine exposure with significant rifabutin exposures and toxicity
Delstrigo^®^ (doravirine/lamivudine/tenofovir disoproxil fumarate)	Doravirine AUC and C_min_ is reduced by 50% and 68% 52	Additional 100 mg of doravirine should be taken 12 hours after Delstrigo^®^ dose

AUC, area under the curve; cART, combined antiretroviral therapy; NRTI, nucleos(t)ide reverse transcriptase inhibitor.

* No studies have been carried out with tenofovir alafenamide (TAF) and rifabutin. However, based on pharmacokinetic studies assessing the effect of rifampicin‐TAF co‐administration on levels of the active metabolite intracellular tenofovir diphosphate (TFV‐DP) 54, it is unlikely that co‐administration of TAF and rifabutin will significantly affect levels of TFV‐DP 25‐*O*‐desacetyl‐rifabutin des‐rifabutin.

There is ongoing accelerated roll‐out of generic integrase strand transfer inhibitor (INSTI)‐based cART in low‐ and middle‐income countries (LMICs). Single/double dose raltegravir (400 to 800 mg bd) and dolutegravir (50 mg bd) are currently recommended doses when used with rifampicin, based on pharmacokinetic and limited clinical trial data 16, 17, 18.

Based upon healthy volunteer studies measuring raltegravir 5 and dolutegravir 19 concentrations, co‐administered with 300 mg once daily (od) rifabutin, no dose changes are recommended 17. Data from the DAWNING study support use of dolutegravir as a second‐line option where it is used in combination with at least one fully active nucleoside reverse transcriptase inhibitor 20. Therefore, the co‐administration of rifabutin with dose unadjusted raltegravir or dolutegravir in first line cART and the co‐administration of rifabutin with dolutegravir in second line cART, would be easier to implement in a programmatic setting and potentially cost saving.

INSTIs bictegravir and elvitegravir 150 mg/cobicistat 150 mg should not be co‐administered with rifabutin due to potential suboptimal antiretroviral levels (see Table 1).

In light of a 37% reduction in AUC, rifabutin doses need to be increased to 450 to 600 mg when co‐administered with the non‐nucleoside reverse transcriptase inhibitor (NNRTI) efavirenz (600 mg od) 21. Twice daily dosing is recommended if NNRTIs rilpivarine or doravarine are to be co‐administered with rifabutin (see Table 1).

Traditionally, second‐line cART has usually included a bPI. In LMICs, most programmatic experience has been to co‐administer with double‐dose lopinavir/ritonavir twice daily (800/200 mg bd) if rifampicin is used. This leads to increases in regimen complexity and pill burden, increased cost 22 and potential significant toxicity 23, 24.

The use of rifabutin allows the use of bPIs without the need to adjust their doses but with the need to modify rifabutin dosage. In a pooled population pharmacokinetic analysis inclusive of individual level data from 13 studies (including one paediatric study), Hennig *et al*. showed that co‐administration of rifabutin in HIV‐TB co‐infected patients on ritonavir‐bPIs increased exposure to rifabutin by 280%, with a disproportionate increase in des‐rifabutin. The model suggested a requirement for a 50% to 67% decrease in rifabutin dose if co‐administered with a ritonavir‐bPI 25. The study concluded that the C_max_ achieved with 150 mg od rifabutin, co‐administered with a ritonavir‐bPI was unlikely to exceed 1 mg/L, a threshold above which has been associated with toxicity 25, 26. Further formulations and dosing regimens would be valuable in order to best replicate the pharmacokinetic profile obtained with dosing of 300 mg od rifabutin alone.

Of note, this analysis lacked data from studies assessing rifabutin‐atazanavir/ritonavir co‐administration and darunavir/ritonavir dosed od. In one small pharmacokinetic study in HIV‐infected TB patients, thrice weekly dosing of rifabutin, co‐administered with daily ritonavir‐boosted atazanavir resulted in suboptimal peak concentrations (C_max_ < 0.5 μg/mL) in 13/16 (81%) 27. In clinical practice, when co‐administering a ritonavir‐bPI with rifabutin 150 mg od, monitoring for toxicity is important as is adherence and consideration of therapeutic drug monitoring in cases of sub‐optimal clinical/bacteriological response to treatment.

## 4 Toxicity and switch option

Frequent (>1% to 10%) toxicities secondary to rifabutin include blood dyscrasias, gastrointestinal side effects, hepatoxicity, rash and polyarthralgia 28, 29, 30. The gastrointestinal tolerability of rifabutin may be ameliorated if co‐administered with food. Rare (≤1%) adverse drug reactions (ADRs) include uveitis and an immunological/flu‐like syndrome 28, 31, 32. Discontinuation of rifabutin secondary to toxicity, varies widely in clinical settings, and is linked with the dose per weight of rifabutin, co‐morbidities and potential drug‐drug interactions for example with azole antifungals and macrolides 32. When comparing rifampicin versus rifabutin (both at 150 and 300 mg od doses), in HIV uninfected patients in a RCT setting, there was no significant difference in ADRs reported 6.

Both the likelihood and mechanism of cross‐reactivity in toxicity profiles for rifampicin and rifabutin are unclear. In a retrospective cohort analysis (n = 221, all HIV uninfected), Chien *et al*. showed that in patients re‐treated with rifabutin after having previous ADRs attributed to rifampicin, there was recurrence of the following ADRs: arthralgia 3/5 (60%), dermatological events 19/82 (23%), cholestasis 2/23 (9%), severe hepatitis 2/23 (9%) and gastrointestinal intolerance 3/55 (5%). There was new onset flu‐like syndrome and neutropaenia in 3% and 6% of patients retreated with rifabutin. In this cohort there were 16/221 (7%) serious rifabutin‐related ADRs. The majority of these (13/16, 81%) were new‐onset neutropaenia, most commonly in women 33. In a sub‐cohort of patients who were switched to rifabutin secondary to ADRs which were categorized as probably/definitely due to rifampicin (n = 39), 72% did not develop a rifabutin‐associated ADR and were able to complete TB therapy with rifabutin. The most common recurrent ADR was dermatological 6/11, (54%). The risk of rifabutin intolerance was ninefold higher (OR 9.3, 95% CI 1.6 to 55) with a previous rifampicin‐associated dermatologic event compared to patients with previous rifampicin‐associated liver injury 34. In a case series (n = 6) of HIV co‐infected rifampicin‐associated drug rash with eosinophilia and systemic symptoms syndrome confirmed on diagnostic re‐challenge, all patients tolerated and completed therapy with rifabutin (450 mg od, co‐administered with efavirenz) 35.

These data support rifabutin as a potential switch option in ADRs attributed to rifampicin, although further larger studies are needed to verify safety results, particularly in HIV co‐infected patients. This is in settings where facilities are present for consecutive and additive drug re‐challenge and close monitoring for drug toxicity and cross‐reactivity. Successful re‐challenge with rifabutin would negate the need to prolong treatment in the absence of a rifamycin as part of short‐course multi‐drug therapy. This would be cost‐saving in terms of clinic visits, personnel and monitoring costs, with public health impact and individual patient benefit.

Although rifabutin is not recommended as first line therapy for latent TB infection (LTBI), it has been used in liver transplant patients, who have experience isoniazid‐induced hepatoxicity and to optimize maintenance of calcineurin inhibitor levels 36. One pilot study (n = 44) for treatment of LTBI in people living with HIV, showed favourable ADR and completion rates comparing three months bi‐weekly rifabutin in combination with isoniazid compared with six months daily isoniazid 37.

Although not teratogenic in animal studies, there are no adequate and well‐controlled study data available on use of rifabutin in pregnant or lactating women to inform of a drug‐related risk.

## 5 Rifampicin‐resistant rifabutin‐susceptible TB

Genotypic resistance to both rifampicin and rifabutin is associated with single nucleotide polymorphisms (SNPs) in the 81‐base pair rifampicin resistance determining region (RRDR) within the *rpoB* gene of *Mycobacterium tuberculosis*. The critical concentration for rifabutin is accepted as 0.5 μg/mL. Some SNPs found in the RRDR, particularly in codon 516 of the *rpoB* gene, although leading to an increase in the minimum inhibitory concentration to rifabutin, are associated with incomplete cross‐resistance to rifampicin and rifabutin 38. Phenotypically determined rifabutin susceptibility in rifampicin‐resistant isolates, as calculated from cross‐resistance studies performed in different geographical cohorts, is estimated at 20% (95% CI 19 to 22; see Table 2). Hence, one in five patients with rifampicin‐resistant TB could benefit from inclusion of rifabutin in their anti‐TB regimen.

**Table 2:** Prevalence of rifabutin sensitivity in rifampicin‐resistant clinical isolates from different geographical cohorts

Population	Ascertainment of Rifabutin susceptibility	Prevalence of Rifabutin susceptibility^a^
Turkey 55	Agar proportions methods and sequencing of *rpoB* gene	6/41 (15%)
South Africa 56	MYCOTB Sensititre plate method and sequencing of *rpoB* gene	51/189 (27%)
South Africa 57	WGS and BACTEC 960 method	WGS 34/149 (23%). Out of these, 32/34 (97%) were confirmed to be susceptible by phenotypic testing
South Africa 39	BACTEC 960 and sequencing of *rpoB* gene	117/349 (33.5%)
Turkey 58	Agar proportions methods	14/52 (26.9%)
Taiwan 59	Agar proportions methods and sequencing of *rpoB* gene	104/800 (13%)
Japan 60	7H9 microbroth dilution method and sequencing of *rpoB* gene	20/98 (20%)
Japan 61	7H9 microbroth dilution method and sequencing of *rpoB* gene	17/93 (18%)
China 62	Microplate alamarBlue and sequencing of *rpoB* gene	52/256 (20.3%)
Belgium 39	BACTEC 480 and 960 and sequencing of *rpoB* gene	29/172 (16.9%)
South Korea 41	Phenotypic (LJ slopes, CC = 20 μg/mL)	31/146 (21%)

CC, critical concentration; LJ, Lowenstein Jensen; WGS, whole genome sequencing.

a Cohorts included had minimum sample size n > 40.

Whitfield *et al*. collated genotypic and phenotypic susceptibility for rifampicin and rifabutin from 2000 MTB isolates. Among 112 *rpoB* SNPs identified, 11 were significantly associated with rifabutin susceptibility and six with rifabutin resistance 39. The 516 GAC→GTC SNP accounted for 70% to 75% of all potentially rifampicin‐resistant rifabutin‐susceptibility from two population‐representative samples, one with high and one with low HIV co‐prevalence 39. This SNP, which is detected by both the Hain MTBDR*plus* line probe assay and Xpert MTB/RIF Ultra molecular beacon assay, could enable accelerated determination of rifampicin‐resistant rifabutin‐susceptible isolates in a programmatic setting. The commercially available validated MYCOTB Sensititre plate method includes rifabutin in its drug panel and yields susceptibility results after a median of 10 days from time of inoculation of cultured strain into the MYCOTB well plates 40. Whole genome sequencing of isolates and clinical samples is becoming more widely available with shorter turnaround times. It enables screening for all known SNPs associated with rifabutin resistance and susceptibility, facilitating SNP‐based phenotypic predictions.

In settings where rifabutin susceptibility testing is available for the construction of individualized regimens, the inclusion of rifabutin in the treatment of patients with rifampicin‐resistant rifabutin‐susceptible strains, could improve bactericidal and sterilizing activity of the regimen, and hence, long‐term outcomes.

Treatment outcome data for use of rifabutin in rifampicin‐resistant TB, particularly in HIV co‐infected patients, is sparse. Jo *et al*. showed in a South Korean cohort of 14 patients with rifampicin‐resistant rifabutin‐susceptible TB, of whom 10 were extensively drug resistant (XDR)‐TB, treatment with rifabutin led to achievement of treatment cure/completion achieved in 12/14 (85.7%). This was significantly better than outcomes in the comparator rifabutin‐resistant TB group, in which only 22/42 (52.4%) achieved treatment completion/cure (*p* = 0.03) 41. Pretet *et al*. assessed the efficacy and tolerability of rifabutin (450 to 600 mg od), along with a fluoroquinolone‐containing regimen in the treatment of rifabutin‐susceptible multidrug resistant (MDR) TB. Culture conversion at 12 months was 14/23 (61%) while 4/39 (10%) experienced ADRs, requiring discontinuation of treatment 42. Whitfield *et al*. reported cure in 13/17 (76%) of patients with rifampicin‐resistant TB (five XDR‐TB, one pre‐XDR, ten rifabutin susceptible). In a cohort of 76 patients with rifampicin‐resistant rifabutin‐susceptible TB, favourable outcomes were achieved in 42/52 (81%) of patients who received rifabutin and 15/24 (63%) of those that did not. In multivariate analysis, rifabutin was associated with an adjusted odds ratio of a favourable outcome of 9.8 (95% CI 1.65 to 58.37) 43. These results warrant future RCTs assessing utility of rifabutin as a replacement for rifampicin as a key sterilizing drug in a rifampicin‐resistant TB regimen or as a companion drug minimizing treatment failure due to acquired drug resistance against core drugs such as fluoroquinolones or bedaquiline 44, 45. The effect of steady‐state rifabutin on bedaquiline pharmacokinetic parameters is significantly less with rifabutin than rifampicin, with a 10% drop in AUC_0‐∞_ with rifabutin versus 44% drop in AUC_0‐∞_ with rifampicin in a pharmacokinetic study with predominantly Caucasian participants 46.

## 6 Cost considerations

We have compared the cost of using rifabutin to rifampicin in a range of TB treatment scenarios. The current price for rifabutin per 150 mg tablet is $0.94 47. Estimated generic prices for rifabutin regimens were calculated using a previously validated cost estimation algorithm 48, 49. Per‐kilogram price of active pharmaceutical ingredient exported from India was collected from the www.infodriveindia.com database. Excipients, formulation costs, packaging, tax and a 10% profit margin were added to calculate the estimated generic price. We looked at a range of utilized rifabutin dosage scenarios. For comparison, the generic cost of a six‐month course of first line anti‐TB treatment including rifampicin 600 mg od in a fixed dose combination is $28.56. The estimated generic cost for six months of first line anti‐TB regimen inclusive of rifabutin 150 mg ×3/week and 150 mg od is $42.33 and $81.54 respectively. The estimated generic cost for six months of first line anti‐TB treatment including rifabutin 300 mg od is $146.88. Of note, widespread use of rifabutin in fixed dose combinations with other anti‐TB drugs, may further decrease production costs. Also, although rifampicin is currently cheaper, the additional dolutegravir that would be needed in HIV co‐infected patients would offset this.

## 7 Conclusions

The favourable pharmacokinetic profile of rifabutin, compared with rifampicin, makes it an attractive choice for concurrent use with several first and second line cART regimens being rolled out in high burden settings. This facilitates the commencement of cART and the maintenance of virological suppression during TB treatment, within routine care settings, with minimal dose changes and toxicity. There is a need for specific studies to verify that some of the presumed salutary interactions of rifabutin with cART regimens result in long term success rates compared to rifampin‐based regimens. Rifabutin has incomplete cross‐reactivity in toxicity profiles for rifampicin, allowing its potential substitution in cases of rifampicin‐associated ADRs. One in five patients with rifampicin‐resistant TB could benefit from the addition of rifabutin to their anti‐TB regimen. Its estimated generic cost should encourage buy‐in from government and non‐governmental organizations to facilitate generic manufacture and widespread accessibility.

## Competing interests

None declared.

## Authors’ contributions

NR wrote the initial draft of the publication. MB modelled the estimated generic price calculations. All authors critically reviewed and approved the final version of the publication.
